# Primary Cutaneous Anaplastic Large Cell Lymphoma Mimicking Pyoderma Gangrenosum: An Atypical Presentation and Diagnostic Challenge

**DOI:** 10.7759/cureus.108047

**Published:** 2026-04-30

**Authors:** Mossaab El Mousadik, Tahar Tazi, Monsif Fadi, Najat Lamalmi, Nouama Bouanani

**Affiliations:** 1 Department of Clinical Hematology and Cellular Therapy, Centre Hospitalier Universitaire (CHU) Souss-Massa, Agadir, MAR; 2 Department of Hematology, Cheikh Khalifa International University Hospital, Mohammed VI University of Health Sciences (UM6SS), Casablanca, MAR; 3 Department of Pathology, Cheikh Khalifa International University Hospital, Casablanca, MAR; 4 Department of Oncopathology, Cancer Biology and Environment Laboratory, Center for Doctoral Studies (CEDoc), Mohammed VI Faculty of Medicine, Mohammed VI University of Health Sciences (UM6SS), Casablanca, MAR; 5 Department of Pathology, Mohammed VI University of Health Sciences (UM6SS), Casablanca, MAR; 6 Department of Hematology, Faculty of Medicine, Mohammed VI University of Health Sciences (UM6SS), Casablanca, MAR

**Keywords:** anaplastic large cell lymphoma, cutaneous t cell lymphoma, pc-alcl, primary cutaneous lymphoma, pyoderma gangrenosum

## Abstract

Primary cutaneous anaplastic large cell lymphoma (PC-ALCL) is a rare subtype of cutaneous T-cell lymphoma that is known for its CD30-positive atypical large lymphoid cells. PC-ALCL usually presents as solitary nodules and has both clinical and histological features that could mimic other cutaneous lymphomas, which can lead to misdiagnosis. We report the case of a 62-year-old patient presenting with chronic ulcers whose condition at first mimicked pyoderma gangrenosum (PG), but eventually turned out to be PC-ALCL following immunohistochemical investigations. Radiological assessment demonstrated suspected regional lymph node involvement. The patient responded partially to cyclophosphamide, doxorubicin, vincristine, etoposide, and prednisone (CHOEP) chemotherapy and achieved a complete metabolic response after second-line chemotherapy consisting of dexamethasone, high-dose cytarabine, and carboplatin (DHAC) followed by BEAM-ASCT consolidation. This case highlights diagnostic and therapeutic challenges in atypical presentations of PC-ALCL.

## Introduction

Primary cutaneous anaplastic large cell lymphoma (PC-ALCL) is a cutaneous T-cell lymphoma subtype characterized by the proliferation of large CD30-positive atypical lymphoid cells. PC-ALCL accounts for approximately 8% of all primary cutaneous lymphomas, and it belongs to the spectrum of primary cutaneous CD30-positive lymphoproliferative disorders, which also includes lymphomatoid papulosis [1]. PC-ALCL typically presents as solitary nodules in approximately 80% of cases, while multifocal nodules or tumors occur in about 20% of cases [2]. Despite its generally indolent course, with 10-year survival rates exceeding 90%, relapses are frequent [3]. Atypical clinical presentations can occur and may lead to diagnostic challenges; more particularly, ulcerated lesions may mimic infectious or inflammatory dermatoses such as pyoderma gangrenosum, resulting in delayed diagnosis and inappropriate initial management [4]. Histopathological evaluation using exhaustive and concise immunohistochemistry panels remains essential for establishing the diagnosis and distinguishing PC-ALCL from other cutaneous lymphomas. Extracutaneous involvement occurs in 10-15% of cases [1], and it may impact both staging and therapeutic decision-making, as localized lesions are treated with surgery or local radiotherapy, while multifocal disease or extensive cutaneous lesions are recommended to be treated with systemic therapies such as methotrexate, brentuximab vedotin or in some refractory situations with multiagent chemotherapy [5]. We report a diagnostically challenging case of PC-ALCL presenting as a chronic ulcerative lesion mimicking pyoderma gangrenosum, highlighting the importance of repeated clinicopathological correlation, comprehensive immunohistochemical evaluation, and cautious staging in cases of atypical or progressive ulcerative presentations.

## Case presentation

A 62-year-old man presented with a two-year history of recurrent cutaneous nodules located on the posterior side of the right upper leg (RUL) region. These lesions evolved in a relapsing-remitting pattern, characterized by episodes of ulceration and fistulization with partial regression following empiric antibiotic therapy.

Six months prior to consulting, the patient experienced progressive worsening of the lesions, with the development of a large ulcerative painful plaque extending from the posterior RUL to the ipsilateral gluteal region, followed by a progressively evolving right inguinal mass, associated with significant weight loss and subjective fever.

**Figure 1:**
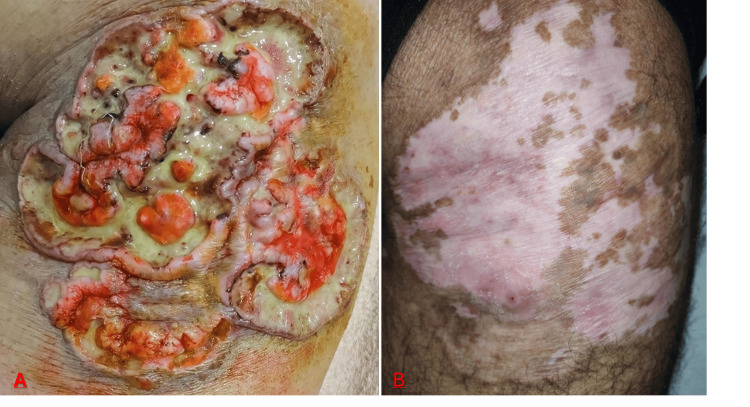
Cutaneous Lesion Evolution (A) Large ulcero-necrotic plaque with purulent exudate, fibrinous slough, and undermined violaceous borders on the posterior right upper leg. (B) Complete healing after second-line treatment with re-epithelialization and residual hypopigmented scarring.

On physical examination, a large, ulcerated plaque measuring approximately 16 × 12 centimetres was observed on the posterior side of the RUL region. The lesion showed an irregular ulcero-necrotic base with purulent exudate and yellow fibrinous slough, with serpiginous violaceous undermined borders, as well as surrounding induration and erythematous halo (Figure 1).

Palpation revealed firm right inguinal lymphadenopathy. Laboratory findings revealed systemic inflammation, with neutrophilic leukocytosis and elevated C-reactive protein, while procalcitonin and lactate dehydrogenase levels were within the normal range. No microbiological evidence of infection was identified on direct bacteriological examination or culture (Table 1).

**Table 1:** Initial laboratory findings The reference ranges in this table are based on commonly accepted adult clinical laboratory standards and may vary depending on institutional and regional laboratory practices.

Parameter	Result	Reference Range
Hemoglobin (Hb)	13.1 g/dL	13.0–17.0 g/dL (men)
White blood cells (WBC)	10,640/mm³	4,000–10,000/mm³
Neutrophils (absolute)	8,510/mm³	1,500–7,500/mm³
Lymphocytes (absolute)	1050/mm³	1,000–4,000/mm³
Monocytes (absolute)	900/mm³	200–1000/mm³
Platelets	251,000/mm³	150,000–400,000/mm³
C-reactive protein (CRP)	70 mg/L	<5 mg/L
Procalcitonin (PCT)	0.04 ng/mL	<0.1 ng/mL
Lactate dehydrogenase (LDH)	228 U/L	85–230 U/L
HIV 1,2 serology	Negative	Negative

**Figure 2:**
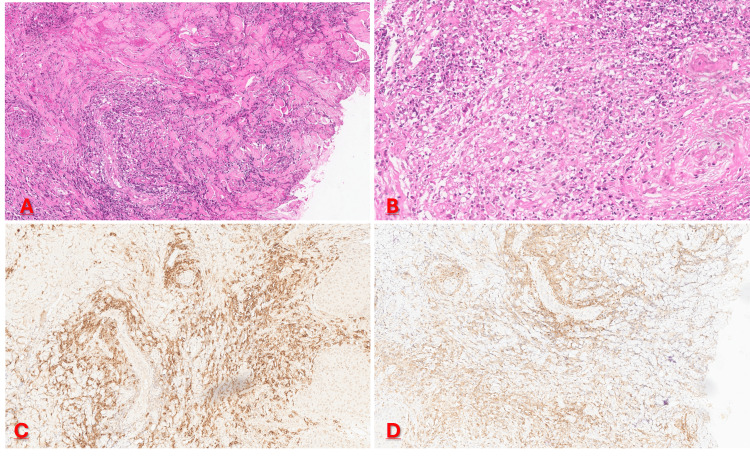
Histopathological and immunohistochemical findings from skin biopsy. (A) Hematoxylin and eosin stain (×20) showing an acanthotic epidermis with irregular hyperplasia and focal ulceration, overlying an edematous and congestive dermis with dense inflammatory infiltrate. (B) Hematoxylin and eosin stain (×40) demonstrating a polymorphous dermal infiltrate rich in neutrophils with perifollicular distribution and prominent angiogenesis with thickened vascular walls and angiocentric lymphocytes. (C) Immunohistochemistry for CD30 (×20) showing diffuse strong positivity in the majority of infiltrating atypical cells. (D) Immunohistochemistry for CD4 (×10) demonstrating diffuse positivity consistent with a T-cell phenotype.

Initial histopathological examination of a skin biopsy obtained from the ulcer edge of the lesion described an ulcerated epidermis with irregular hyperplasia. The dermis was infiltrated by a dense inflammatory infiltrate rich in neutrophils and lymphocytes, with perivascular cuffing and marked angiogenesis. The infiltrate extended into the subcutaneous tissue and included plasma cells and histiocytes. These findings were initially suggestive of pyoderma gangrenosum. However, B symptoms, progressive cutaneous lesions, and suspected nodal involvement raised clinicopathological discordance and suggested a neoplastic process. This prompted re-reading of the biopsy specimen, which revealed large atypical cells with strong expression of CD30, CD3, and CD4, consistent with a T-cell phenotype, and negative for CD8, CD20, and CD15, with heterogeneous CD5 expression. The proliferation index assessed by Ki-67 was approximately 60%, while anaplastic lymphoma kinase (ALK) and epithelial membrane antigen (EMA) were negative. These findings supported the diagnosis of primary cutaneous anaplastic large cell lymphoma (Figure 2).

**Figure 3:**
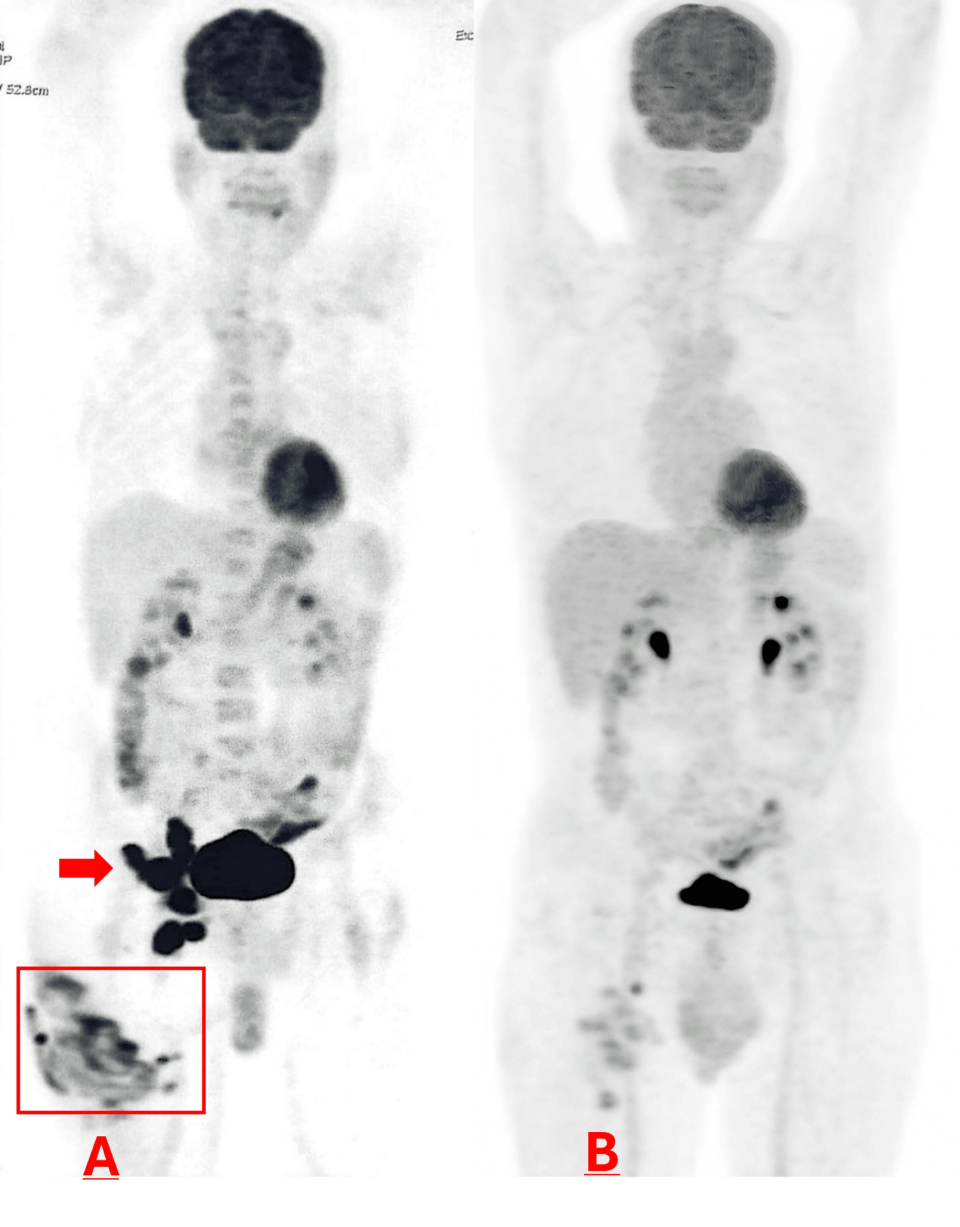
Positron emission tomography/computed tomography findings before and after treatment. (A) Coronal image demonstrating a hypermetabolic right superficial inguinal nodal conglomerate (SUVmax 19.10) and a right internal iliac lymph node (SUVmax 15.08) (arrow). Focal cutaneous and subcutaneous hypermetabolism in the posterior proximal right thigh (SUVmax 9.07; square). (B) Coronal image at follow-up after second-line treatment demonstrating complete metabolic response.

Positron emission tomography-computed tomography (PET-CT) showed intense hypermetabolism of the cutaneous lesion with hypermetabolic right inguinal and internal iliac lymph nodes. As the TNM classification requires histopathological confirmation for nodal staging and no biopsy was performed, nodal status was classified as Nx. The disease was staged as T2b Nx M0 (Figure 3).

The patient received six cycles of the CHOEP regimen administered every 21 days, consisting of cyclophosphamide 750 mg/m² (day 1), doxorubicin 50 mg/m² (day 1), vincristine 1.4 mg/m² (day 1), etoposide 100 mg/m² (days 1-3), and prednisone 100 mg/day (days 1-5). End-of-treatment PET-CT showed a partial metabolic response, with residual uptake at the primary lesion site corresponding to a Deauville score of 4 [6]. Second-line treatment with DHAC was initiated, consisting of dexamethasone 40 mg/day (days 1-4), cytarabine 2 g/m² every 12 hours (day 2), and carboplatin AUC 5 (day 2), leading to complete metabolic remission after two cycles, with a Deauville score of 1 on PET-CT. Consolidation with a BEAM conditioning regimen consisted of carmustine 300 mg/m² (day -7), etoposide 100 mg/m²/12 hours and cytarabine 100 mg/m²/12 hours (day -6 to day -3); and melphalan 140 mg/m² (day -2), followed by autologous stem cell transplantation (ASCT). This resulted in sustained complete remission at 5 months following ASCT.

## Discussion

The present case reports an unusual presentation of primary cutaneous anaplastic large cell lymphoma (PC-ALCL), mimicking the clinical and histopathological features of the ulcerative form of pyoderma gangrenosum (PG).

Primary PG is an uncommon neutrophilic dermatosis characterized by rapidly progressive painful skin ulcers, with undermined violaceous borders and surrounding erythema. Its prevalence is low, with only a few cases per million person-years. Although frequently associated with autoimmune and autoinflammatory diseases, its exact pathophysiology remains unclear. PG is a diagnosis of exclusion, requiring the elimination of conditions such as cutaneous lymphomas, squamous cell carcinoma, infections, and vascular ulcers [7]. PC-ALCL typically presents as a solitary or localized nodule [1,2]. In contrast, our patient developed a large necrotic ulcer resembling PG [4].

Histological examination of PG shows dense neutrophilic infiltration with a perivascular lymphocytic component at the ulcer edge [7]. Similar findings in our patient’s biopsy led to the initial misdiagnosis. However, tumor progression, systemic symptoms, and lymphadenopathy raised suspicion of an alternative diagnosis. Immunohistochemistry is essential for diagnosing PC-ALCL and should include markers such as CD3, CD4, CD8, CD20, CD30, CD56, ALK, and PAX5 [1]. In our case, atypical large cells were CD30-positive with a T-cell phenotype (CD3+, CD4+) and a high proliferation index (Ki-67 > 60%), while anaplastic lymphoma kinase (ALK) was negative, supporting the diagnosis of PC-ALCL. Although typically ALK-negative, rare PC-ALCL ALK-positive cases have been reported [8].

Positron emission tomography-computed tomography (PET-CT) demonstrated increased metabolic activity in regional lymph nodes, a nonspecific finding that may also occur in inflammatory conditions, particularly in ulcerated or infected lesions [9]. As the TNM classification requires histological confirmation for nodal staging [10] and no lymph node biopsy was performed, nodal status should be classified as Nx. Biopsy was deferred to avoid delaying management in the setting of an aggressive presentation with a painful ulcer, B symptoms, and declining general condition. Given the potential delay associated with histopathological confirmation in routine practice, clinical and PET-CT findings were used to guide management. Nodal involvement was considered suggestive of disease progression, an interpretation supported by the subsequent response to systemic therapy.

PC-ALCL generally has a favorable prognosis [3], though extracutaneous involvement, widespread disease, and relapses are associated with poorer outcomes [11]. In this patient, adverse prognostic factors included extensive cutaneous involvement, systemic symptoms, and inadequate response to initial therapy.

Therapeutic strategy depends on disease extent. Localized lesions are managed with surgical excision or radiotherapy, whereas advanced or extracutaneous disease requires systemic therapy. In our case, extensive cutaneous involvement, regional lymphadenopathy, and systemic symptoms suggested aggressive disease, supporting prompt systemic treatment. ESMO guidelines indicate that multi-agent chemotherapy may be considered in patients with extracutaneous involvement or rapidly progressive disease [3,5]. Brentuximab vedotin is an effective option for CD30-positive cutaneous T-cell lymphomas, with superior response rates compared to methotrexate or bexarotene in the ALCANZA trial (ORR4 75% vs 20%) [12]. However, it was not used due to limited availability and cost. Given the aggressive presentation and the lower efficacy of methotrexate compared with brentuximab vedotin, systemic multi-agent chemotherapy was favored to achieve rapid disease control. This approach resulted in complete metabolic remission after second-line treatment. Consolidation with autologous stem cell transplantation (ASCT) was subsequently performed and may be considered in selected high-risk cases [13].

This case has several limitations. First, histological confirmation of lymph node involvement was not obtained, and nodal staging was therefore based on imaging findings. In addition, brentuximab vedotin, a recommended targeted therapy, was not used due to limited availability and cost, which may have influenced therapeutic decision-making. As a single case report, the findings may not be generalizable and should be interpreted in the context of existing literature.

## Conclusions

The current case describes an atypical presentation of primary cutaneous anaplastic large cell lymphoma (PC-ALCL). This observation emphasizes the need for re-evaluation of biopsy results in cases of discrepancy between clinical findings and histology and highlights the importance of repeated tissue sampling with comprehensive immunohistochemical analysis in atypical or non-healing ulcerative lesions. Importantly, this case illustrates how initial misdiagnosis and delayed recognition may contribute to disease progression and the emergence of systemic features. In addition, it demonstrates the challenges of distinguishing between primary cutaneous lymphoma and systemic disease in clinical practice. Finally, the aggressive clinical course observed in this patient suggests that intensified systemic therapy may be considered in selected high-risk cases, highlighting the need for further research to define prognostic factors, including clinical features, that may help identify patients most likely to benefit from more aggressive therapeutic strategies.
